# Supplementary material for: Fate and propagation of endogenously formed Tau aggregates in neuronal cells
Source: EMBO Mol Med. 2020 Nov 12;12(12):e12025. doi: 10.15252/emmm.202012025 (PMC7721367; doi:10.15252/emmm.202012025)
Supplement: Supplementary file 1 — Expanded View Figures PDF [file EMMM-12-e12025-s001.pdf]

Expanded View Figures

Figure EV1. Spreading of K18-ATTO 594 fibrils and of DID.

A Quality control of K18 fibrils. After incubation in the presence (+) or absence (−) of heparin, fibrils were purified by ultracentrifugation. Supernatant (SN) and pellet were controlled by SDS–PAGE (18%) followed by Coomassie staining (upper panel), and by thioflavin T assay (graph below) where fluorescence intensity was monitored (Ex 450, Em 510 nm, integration time 200 ms). On the right side of the gel are the apparent molecular weights (kDa), and the white lane indicates that intervening lanes from the same gel have been spliced out. This experiment is representative of three independent preparations of fibrils.

B Uptake of K18 fibrils by cells. Three representative confocal pictures (one Z-stack in the 2D picture, orthogonal views covering 17 μm in 17 stacks) of CAD cells, first challenged with K18-ATTO 594 fibrils, trypsinized 24 h later, and replated for an additional 24 h, in the conditions used for all coculture experiments. White is WGA staining, and red is the fibrils; scale bars are 10 μm.

C Transfer of DiD in CAD cells. Left, quantification by flow cytometry of the relative percentage of DiD-loaded acceptor cells upon treatment with CK666 during the coculture. Data represent the means (+ SD), normalized to non-treated coculture arbitrarily set at 100%, of three independent experiments, with statistical analysis by two-tailed unpaired *t*-test (mean + CK666=120%, ****P* = 0.0008). Right is the same analysis when the cell were cultured in sparse conditions, not favoring direct cell contacts (mean = 45%, three independent experiments, **P* = 0.011).

D Spreading of K18-ATTO 594 fibrils in SH-SY5Y cells. Quantification by flow cytometry of the percentage of K18-ATTO 594-positive acceptor cells after coculture of donor and acceptor cells (total), or culture of acceptor cells with donor-conditioned medium for 24 h (secretion). The total transfer was arbitrarily set at 100%, and cell-to-cell contact transfer was calculated by subtracting secretion transfer from total transfer. Data represent the means (+ SD) of four experiments, with statistical analysis by two-tailed unpaired *t*-test (*****P* = 7.17E−10).

© 2020 The Authors

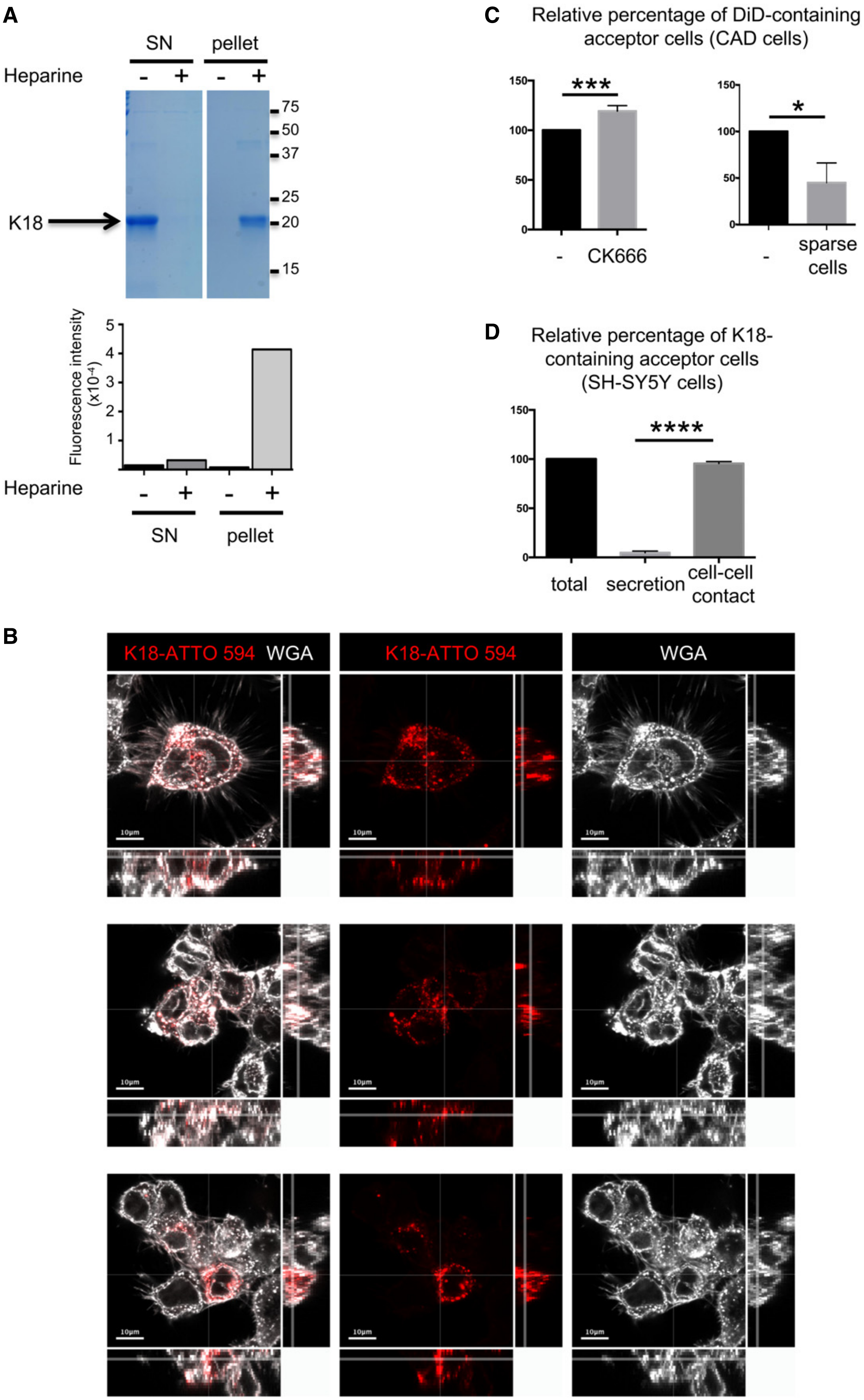

Figure EV1.

 © 2020 The Authors

Figure EV2. Entry of K18 fibrils, seeding, and transfer of full-length Tau in CAD cells.

A CAD cells were transfected with FL Tau 1N4R P301S-YFP encoding plasmid for 6 h, and then challenged or not with non-labeled K18 fibrils (sonicated, with or without Lipofectamine 2000 as indicated) and left o/n before trypsinization and replating for an additional 24 h. Cells were finally fixed, labeled with WGA, and analyzed by confocal microscopy (40× magnification). Pictures of cells containing aggregates are shown for conditions 2 and 3, representative of the results. The arrows point to cells containing fibrils; scale bars are 10 μm.

B The plot shows the percentage with SEM of transfected cells where FL Tau appeared as inclusions (1, 9.8, and 55, respectively, for conditions 1, 2, and 3). Statistically significant differences are compared to the control conditions (1, Lipofectamine without fibrils) by one-way ANOVA and Tukey's *post hoc* test (*****P* = 1.07E-08 for 1 vs 3, 3.65E−10 for 2 vs 3). The efficiency of transfection was 47%, and the numbers of cells containing green aggregates counted were 222, 693, and 526, respectively, for conditions 1, 2, and 3, over three independent experiments.

C Representative confocal pictures (63× with 1.6 zoom, one Z-stack in the 2D picture, orthogonal views covering 5.6 μm in 17 stacks) of CAD cells treated with K18-ATTO 594 fibrils as in (A). Upper left panel is a cell without red or green aggregates, and the three other panels are a cell where FL Tau-YFP is aggregated. Green is FL Tau-YFP, and red is the fibrils; scale bars are 10 μm.

D Below the schematics of the experiment are representative confocal images (40× objective) of donor CAD cells (transfected with FL Tau 1N4R P301S-YFP expression vector), challenged or not with non-labeled K18 fibrils (respectively, second and first lane panels), acceptor cells with conditioned medium from K18-challenged donor cells (acceptor cells + SN, third lane panels), and coculture of donor (with K18) and acceptor cells in the bottom panels. The images are representative Z-stacks, except from the bottom panel which is a maximal projection covering five upper stacks (1.4 μm in total, allowing to visualize TNTs, not attached to the dish). In the merged images, white is WGA, green is YFP, red is mCherry, and nuclei are stained in blue. Arrows point to FLTau puncta inside acceptor cells and the arrowhead shows a green dot inside a TNT, which is indicated with a bracket. Insets are threefold enlargements of the boxed regions in the lower panels. Scale bars are 10 μm.

E Quantification of the percentage of FLTau-positive acceptor cells after coculturing donor and acceptor cells (cells), or culturing acceptor cells with donor-conditioned medium for 24 h (SN). In the scatter dot plot, each symbol is a tile of four fields of acquisition, and bars are means (0.72 and 24.3 respectively) ± SEM. The total number of acceptor cells counted over two independent experiments was 229 for SN and 286 for cell coculture. Statistical analysis was unpaired *t*-test, *P* = 0.0107 (*).

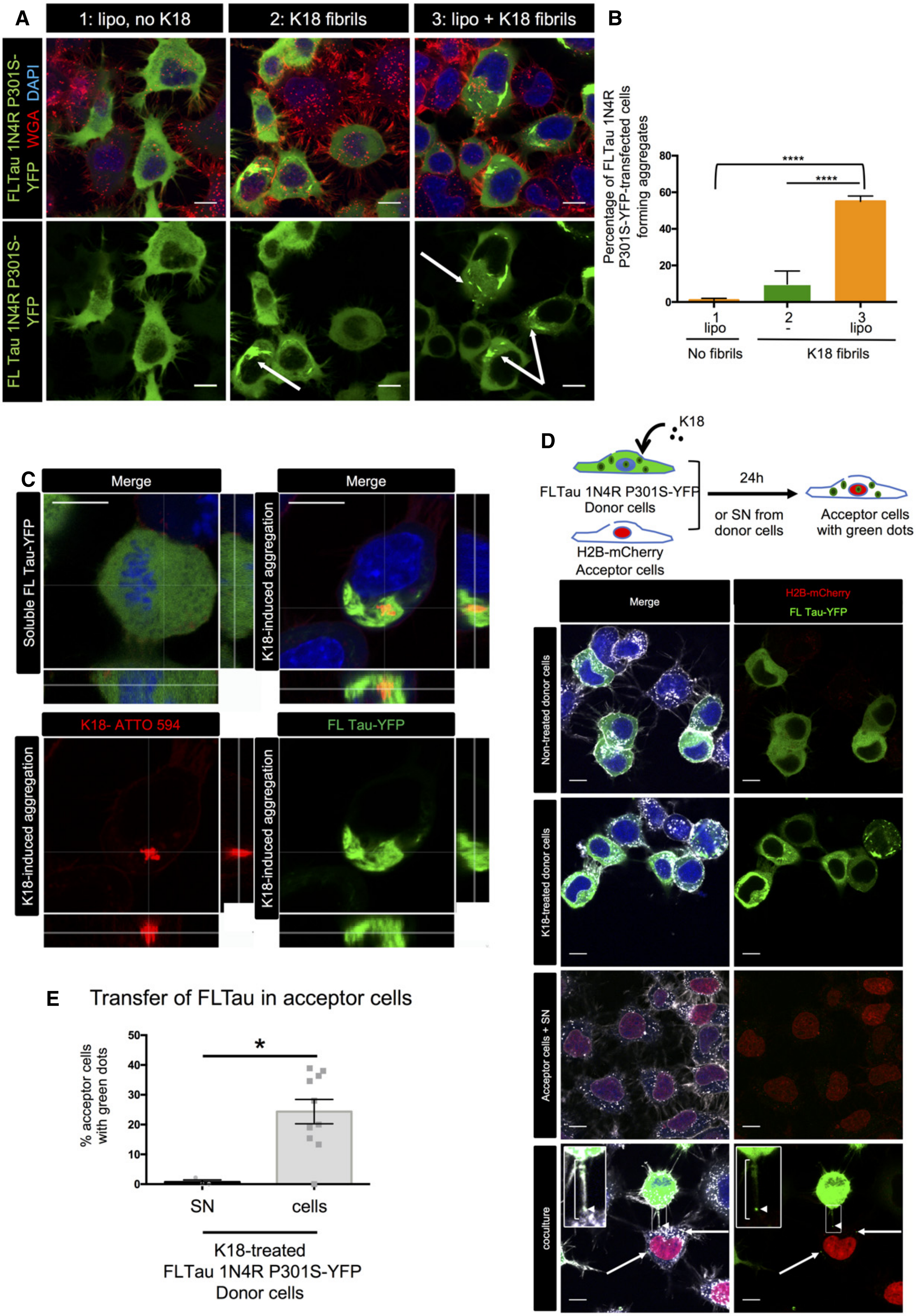

Figure EV2.

 © 2020 The Authors

Figure EV3. Localization of RD-YFP aggregates.

A RD-YFP SH cells were challenged with non-labeled K18 fibrils for 2 days before fixation, saponin permeabilization, and staining with antibodies recognizing, respectively, TOM 20, EEA1, Furin + Giantin, vimentin, alpha-tubulin, or WGA. Representative deconvoluted confocal images are presented, and blue staining is DAPI in the merged pictures; and scale bars are 10 µm.

B Representative confocal images (of three independent experiments) of RD-YFP aggregates overlapping with p62 and ubiquitin, induced either by synthetic K18 fibrils or by AD-derived extracts as indicated on the left. RD-YFP SH cells were challenged with non-labeled K18 fibrils for 2 days or AD XT for 4 days before fixation, saponin permeabilization, and staining with antibodies recognizing all types of ubiquitin chains (Ub) or K63-linked ubiquitin chains (Ub [K63]) and p62 (white). Insets are threefold enlargements of the boxed regions, showing colocalizations of Ub, p62, and RD-YFP; scale bars are 10, 2 µm in insets.

C Ubiquitination of RD-YFP aggregates. Frozen cell pellets of RD-YFP SH cells treated with K18 fibrils and grown for 4 days were thawed on ice and cells were lysed in PBS-Triton X-100 0.05%, and next total extracts (tot) were ultracentrifugated at 100,000 *g* to separate soluble material (S) from pellets (P) corresponding to insoluble material, including aggregates, shown by brackets in the WB of the left panel (4–12% gel in MES buffer, denaturation in 1% Laemmli without reducing agent). Tot and P fractions were loaded again, and the same membrane was blotted consecutively with antibodies against ubiquitin (right panel) and RD-YFP (GFP). Ubiquitinated material and aggregates are shown with brackets, and the arrow points to monomeric RD-YFP. MW (kDa) is indicated for each gel.

D Quantification of the colocalization between RD-YFP and LC3 in various conditions. Cells were treated as in Fig 3A, except that antibody recognizing LC3 was used, and quantifications were performed as in 3B. The graph represents the mean percentage (+ SEM) of green material overlapping with LC3, and the number of cells analyzed was 14, 18, 14, and 24, respectively, for each condition over two experiments. PCC is indicated below the graph. Statistically significant differences were compared to the soluble conditions (one-way ANOVA and Tukey's *post hoc* test, [*****P* = 1.34E−07, 5.79E−10, 8.17E−07]).

E Representative confocal images of RD-YFP SH cells, challenged with non-labeled K18 fibrils for 2 days. LysoTracker (red) was added to the culture 30 min before fixation, and scale bars are 10 µm.

F Representative picture of RD-YDP SH cells, 14 days after challenging with non-labeled fibrils and processed for immunofluorescence as in Fig 3A. Insets are threefold enlargements of the boxed region; white arrows point to colabelings; scale bars are 10 µm.

G Quantification of colocalization of RD-YFP material with p62-positive structures after 7 or 14 days of culture. Confocal pictures were analyzed in 3D with Imaris software, as in Fig 3B. Below the graph (mean percentage with SEM) is indicated the corresponding Pearson's correlation coefficient (PCC). The number of cells analyzed in each condition over three independent experiments was 39, 81, and 65, respectively. Statistically significant differences are compared to the soluble conditions (one-way ANOVA and Tukey's *post hoc* test [*****P* = 6.13E−07 and 7.85E−08 for NT and Baf, respectively]). Note that the differences between agg NT and BafA1 are not significant, for percentage of overlapping and for PCC.

H Analysis of the number of green dots per cell among the population of cells containing RD-YFP aggregates after treatment with bafilomycin A1 or bortezomide. Cells were treated and imaged as described in Fig 3A, and analysis of 63× images was performed using spot detector wizard (scale 3, threshold 80) under Icy software, with a total number of analyzed cells over three independent experiments of 95, 87, and 39 for each condition, respectively. Each analyzed cell is represented on the dot plot, and the bars indicate the means ± SEM (respectively, 106, 120, and 126). Statistical analysis was performed by one-way ANOVA and Tukey's *post hoc* test, and all the pairwise comparisons were not significant (ns). Note that the difference in mean aggregate number compared to Fig 4F is because of the different acquisition conditions (63× instead of 40×).

I RD-YFP aggregates inside a TNT positive for actin (phalloidin labeled, red) and negative for tubulin (white). After treatment with unlabeled K18 fibrils, RD-YFP was plated on coverslips for 24 h and treated with trypsin for 1 min before fixation and immunofluorescence. The 3D view shows eight slices covering 2.45 µm. Below each panel is a twofold magnification of the TNT area. Arrows show aggregates inside actin-only-positive TNTs, and arrowhead shows an aggregate inside an actin and tubulin-positive connection. Scale bars are 10 µm. The average diameter of tubulin and actin-containing connections (as measured based on phalloidin labeling at the level of the aggregate) was 0.6 µm (SD 0.11, *n* = 8), whereas the actin-only-containing connections were thinner (0.40 µm, SD: 0.05, *n* = 4).

© 2020 The Authors

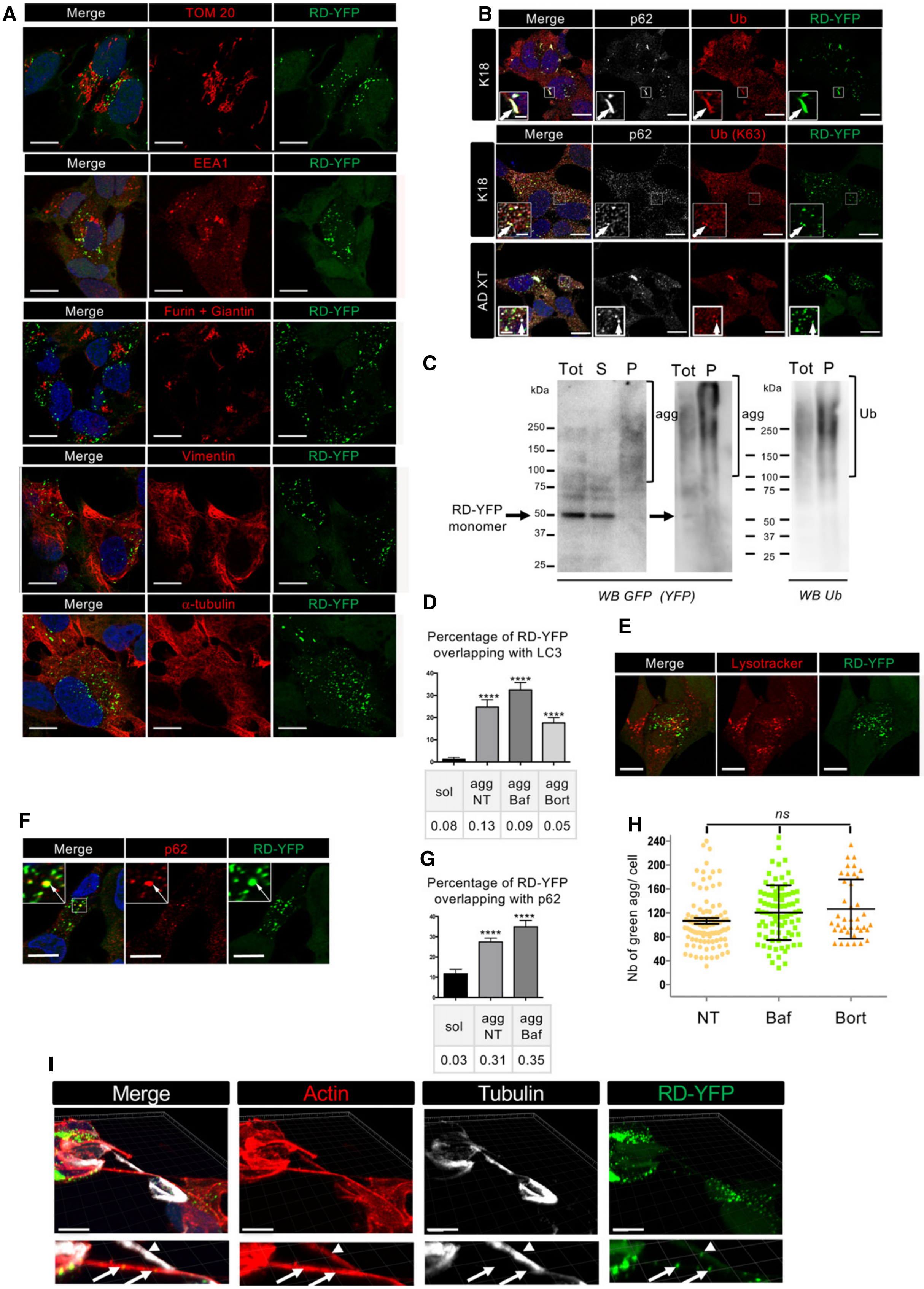

Figure EV3.

 © 2020 The Authors

Figure EV4. Transfer of RD-YFP aggregates in SH-SY5Y derived cells.

A Conditioned medium of cells treated with K18 fibrils is devoid of K18 fibrils. Twenty-four-hour conditioned media (1 ml) of cells (SH-SY5Y or RD-YFP SH) treated or not with K18 as indicated, providing from independent experiments, were ultracentrifuged at 100,000 *g* for 1 h at 4°C. Pellets containing insoluble material were solubilized in 1% SDS-containing Laemmli without reducing agent and analyzed by WB for the presence of K18 (detected with anti-V5 antibody). As positive control, the same volume of fibril-containing medium, collected at the end of the 6-h incubation on cells (i.e., containing fibrils that were not uptaken by cells), was processed the same way (lane 7). The K18 ladder corresponding to the fibrils is indicated by brackets. Right is overexposure of the lanes 1–6 of the membrane.

B Quantification of the percentage of RD-YFP SH acceptor cells with insoluble RD-YFP, depending on the condition (12.5% for donor cells and 0.37% for donor SN) in the experiment described in Fig 4C. Analysis was performed using ICY software, and data represent the number of aggregate-containing cells over the total number of green cells without red nuclei + SEM (the total number of acceptor cells analyzed over two independent experiments was 1,788 for coculture and 1,028 for SN) with statistical analysis by two-tailed unpaired *t*-test (***$P = 0.0006$).

C Visualization of the direct transfer of endogenously formed RD-YFP aggregates to SH-SY5Y cells. Above the pictures is a schematic representation of the experiment. Donor RD-YFP SH cells expressing aggregates obtained 2 days after treatment with non-labeled K18 fibrils were cocultured for 24 h with SH-SY5Y cells first transfected with H2B-mCherry expression vector. The images are Z-stack projections covering nine slices (each 0.43 μm), and similar experiments were performed three times. Transferred RD-YFP aggregate is indicated by the white arrow, and single-channel pictures are grayscale images; and scale bar is 10 μm.

© 2020 The Authors

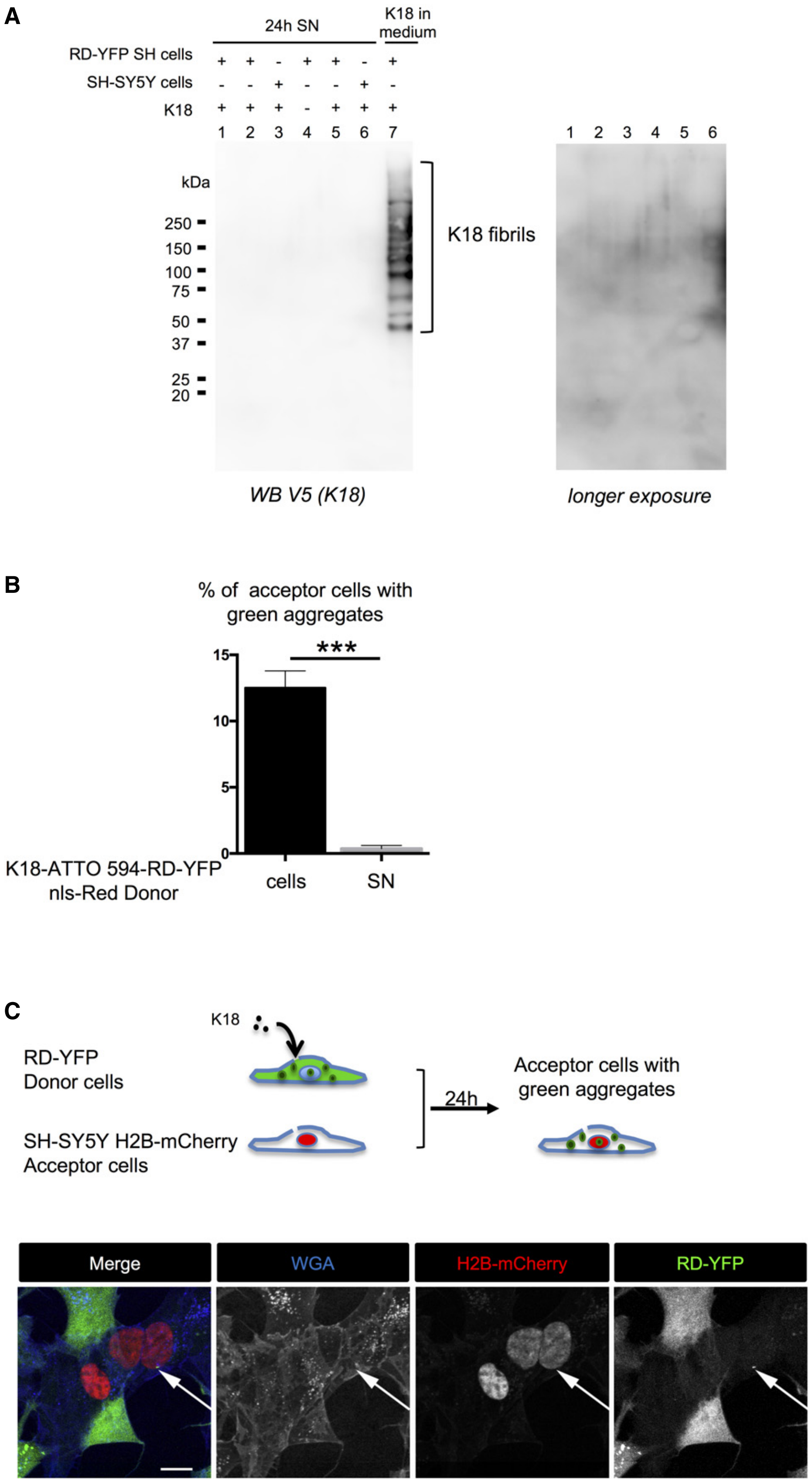

Figure EV4.

 © 2020 The Authors

Figure EV5. Transfer of RD-YFP aggregates from DS9 cells to SH-SY5Y-derived cells.

A Two-day coculture of SH-SY5Y cells expressing RD-YFP and nls-Red (acceptor cells) with DS9 cells (donor cells). Below the schematic representation of the experiment are representative confocal images showing maximum intensity projections of six z-slices (covering 2 μm of thickness). In the merged images, white is WGA labeling, green is RD-YFP, red is nls-Red nuclei, and nuclei are stained in blue with DAPI; scale bars are 10, 5 μm in the enlarged boxes. Below each image are twofold enlargements of the corresponding boxed areas.

B Quantification of experiment described in (A), giving the percentage of converted nls-Red-expressing RD-YFP SH cells after coculture with DS9 cells or SN (donor cells or donor SN). Analysis was performed using ICY software, data represent the number of converted acceptor cells over the total number of nls-Red-expressing RD-YFP SH cells + SEM, and means are 3.04 (donor cells) and 0.03 (donor SN). The total number of RD-YFP SH cells analyzed over three independent experiments was 1,923 for coculture and 1,654 for SN, statistical analysis by two-tailed unpaired *t*-test (***P* = 0.0075).

C Representative confocal image showing a TNT connecting two DS9 cells (green arrow) or connecting one RD-YFP nls-Red SH cell and one DS9 cell (white arrow). To improve visualization of the TNTs, cells were incubated for 1 min with trypsin just before PFA fixation.

D Mean number of green dots per cell ± SEM among the population of cells containing green aggregates in the experiments described in (A). Cell counts were performed over three independent experiments using spot detector wizard under Icy software (scale 1), with a total number 30 acceptor cells analyzed. The scatter dot plot shows each cell as a circle, and the mean (19.7) ± SEM to compare to Fig 4F.

E Visualization of the direct transfer of native RD-YFP aggregates from DS9 to SH-SY5Y cells. Above the pictures is a schematic representation of the experiment. Donor DS9 cells were cocultured for 24 h with SH-SY5Y cells first transfected with mCherry expression vector. Transferred RD-YFP aggregate is indicated by the white arrow, and white is WGA in merge; scale bars are 10 μm.

© 2020 The Authors

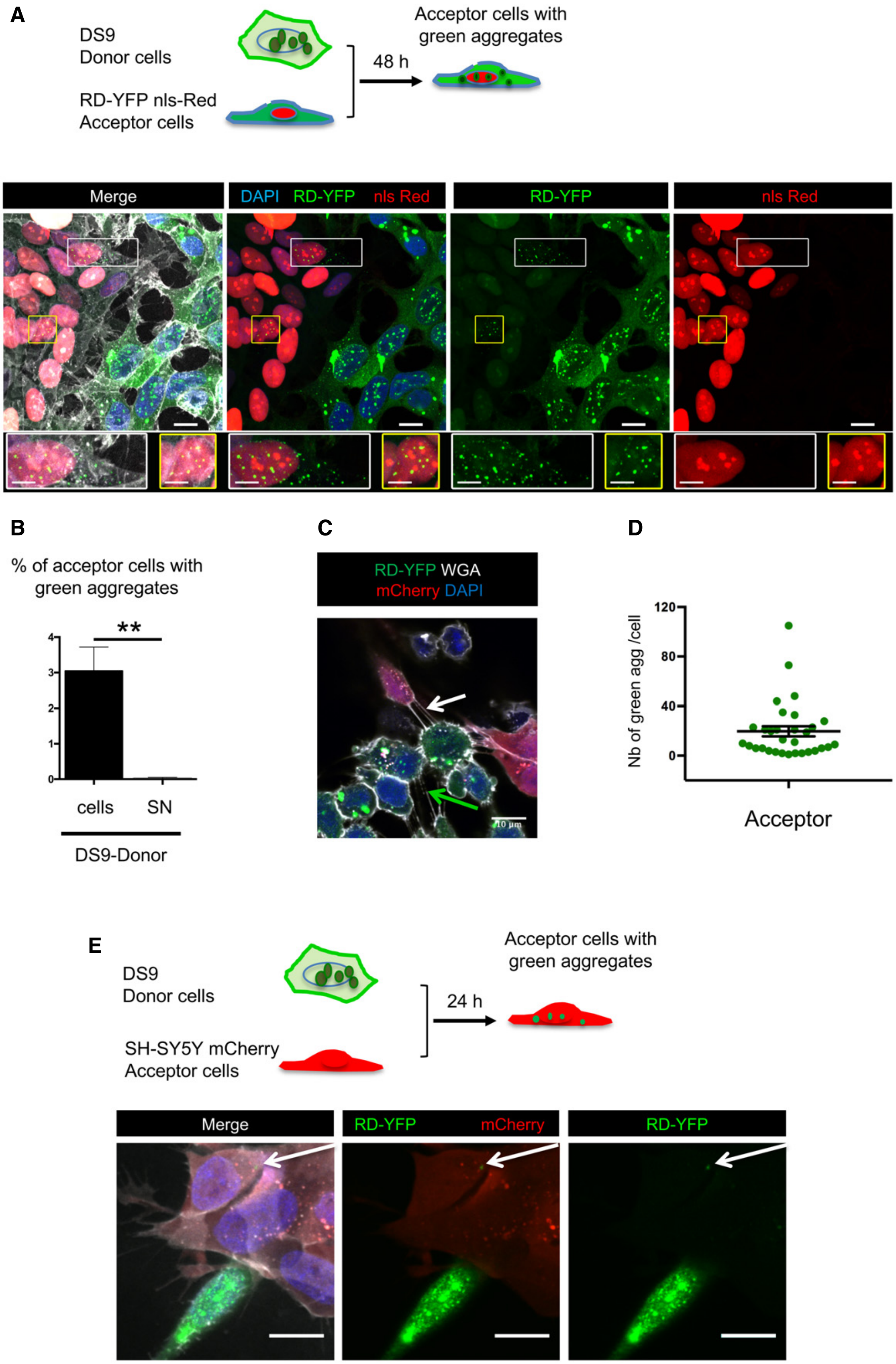

Figure EV5.

 © 2020 The Authors

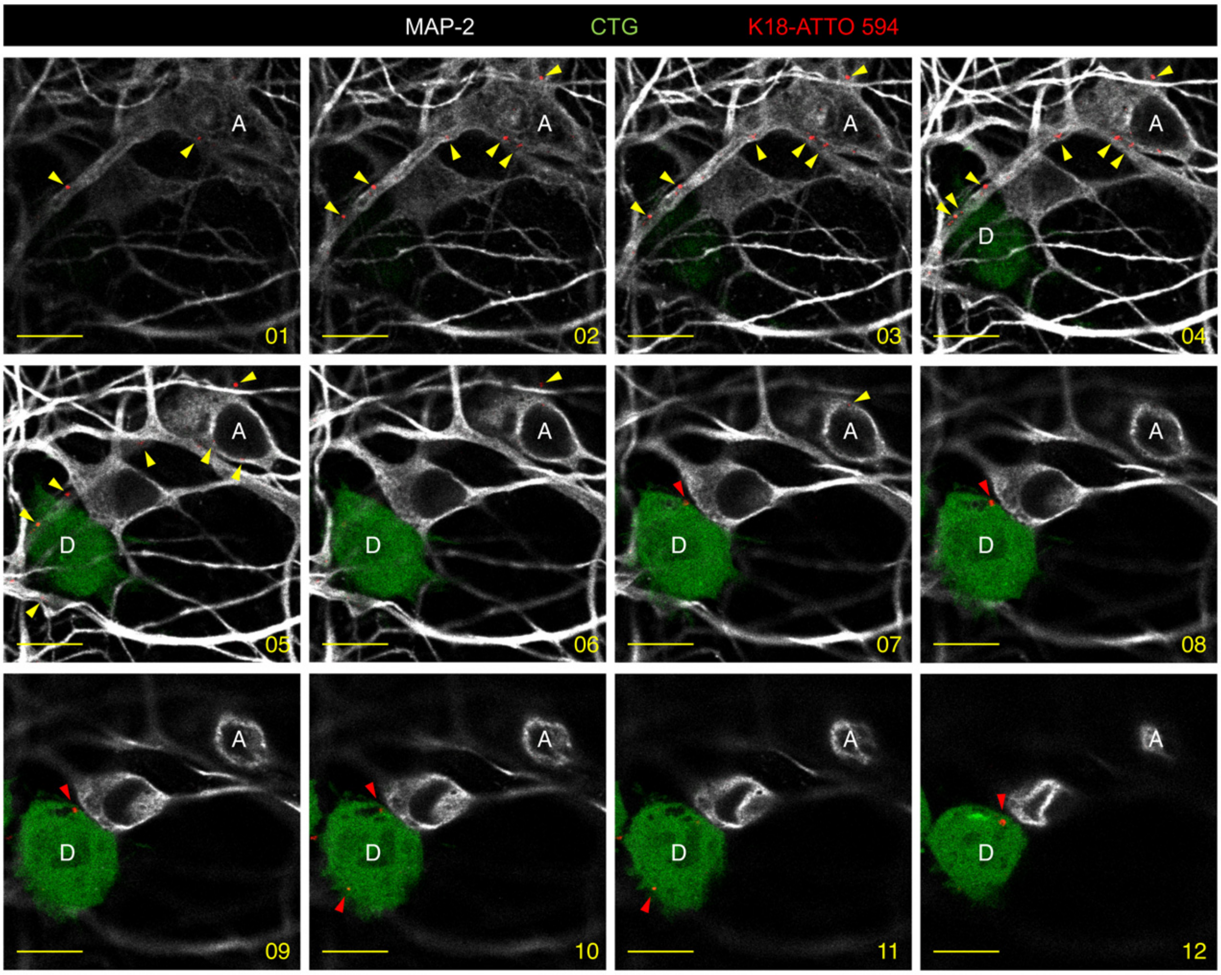

Figure EV6. Transfer of K18-ATTO 594 aggregates from CAD cells to primary neurons.

Unstacked Z-stack images of donor CAD cells and acceptor primary cortical neurons after 24h in coculture allow appreciating that CAD cells (CTG) and neurons (MAP-2) are on different spatial planes. The images of the series cover a range from 0.33 to 4.02 μm (numbered 1–12), with 0.33-μm plane thickness. While red arrowheads point to Tau puncta in donor cells (D), and yellow arrowheads point to Tau puncta detected in the cell body and neurites of an acceptor neuron (A). Scale bars represent 10 μm.
