# Supplementary material for: Fate and propagation of endogenously formed Tau aggregates in neuronal cells
Source: EMBO Mol Med. 2020 Nov 12;12(12):e12025. doi: 10.15252/emmm.202012025 (PMC7721367; doi:10.15252/emmm.202012025)
Supplement: Supplementary file 2 — Movie EV1 [file EMMM-12-e12025-s002.zip › zip movie EV1/Movie EV1 legend.docx]

Movie EV1: DS9 cell culture. DS9 cells stably expressing aggregates of Tau RD-YFP over a 50-hour period monitored by IncuCyte (20x objective) set to acquire images every 30 minutes (video at 15 fps). Green channel (RD-YFP) (Excitation 440-480 nm, 400 ms) is shown. The frame of the video is a square of 200 μm side length.
