# Supplementary material for: Fate and propagation of endogenously formed Tau aggregates in neuronal cells
Source: EMBO Mol Med. 2020 Nov 12;12(12):e12025. doi: 10.15252/emmm.202012025 (PMC7721367; doi:10.15252/emmm.202012025)
Supplement: Supplementary file 4 — Movie EV3 [file EMMM-12-e12025-s004.zip › zip movie EV3/Movie EV3 legend.docx]

Movie EV3: RD-YFP SH cells upon exposure to synthetic red fibrils. RD-YFP SH cells, which express soluble RD-YFP, over a 70-hour period from the addition of K18-ATTO574 fibrils (video at 10 fps). Cells were plated, treated with K18-ATTO 594 fibrils at 1 μM (time 0) and placed into the IncuCyte incubator for six hours and next kept in culture for an additional 60 hours after medium change. IncuCyte (20x objective) was set to acquire images every 30 minutes, green channel (Excitation 440-480 nm, 400 ms) and red (excitation 565-605 nm, 800 ms) channels are shown, 3% spectral un-mixing was applied to the pictures. The frame of the video is a square of 200 μm side length.
