# Supplementary material for: Fate and propagation of endogenously formed Tau aggregates in neuronal cells
Source: EMBO Mol Med. 2020 Nov 12;12(12):e12025. doi: 10.15252/emmm.202012025 (PMC7721367; doi:10.15252/emmm.202012025)
Supplement: Supplementary file 5 — Movie EV4 [file EMMM-12-e12025-s005.zip › zip movie EV4/Movie EV4 legend.docx]

Movie EV4: non-treated RD-YFP SH cells, control to movie 5. RD-YFP SH cells, which express soluble RD-YFP, over a 70-hour period (video at 10 fps). IncuCyte (20x objective) was set to acquire images every 30 minutes, green channel (Excitation 440-480 nm, 400 ms) is shown. The frame of the video is a square of 200 μm side length.
