# Supplementary material for: Fate and propagation of endogenously formed Tau aggregates in neuronal cells
Source: EMBO Mol Med. 2020 Nov 12;12(12):e12025. doi: 10.15252/emmm.202012025 (PMC7721367; doi:10.15252/emmm.202012025)
Supplement: Supplementary file 6 — Movie EV5 [file EMMM-12-e12025-s006.zip › zip movie EV5/Movie EV5 legend.docx]

Movie EV5: RD-YFP SH cells upon exposure to AD-derived brain extract. RD-YFP SH cells, which express soluble RD-YFP, over a 70-hour period from the addition of AD-derived fibrils (video at 10 fps). Cells were plated, treated with fibrils (time 0) and placed into the IncuCyte incubator for six hours and next kept in culture for an additional 60 hours after medium change. IncuCyte (20x objective) was set to acquire images every 30 minutes, green channel (Excitation 440-480 nm, 400 ms) is shown. The frame of the video is a square of 200 μm side length.
